# Promising Activities of Marine Natural Products against Hematopoietic Malignancies

**DOI:** 10.3390/biomedicines9060645

**Published:** 2021-06-05

**Authors:** Assunta Saide, Sara Damiano, Roberto Ciarcia, Chiara Lauritano

**Affiliations:** 1 Marine Biotechnology Department, Stazione Zoologica Anton Dohrn, Villa Comunale, 80121 Napoli, Italy; assunta.saide@szn.it; 2 Department of Veterinary Medicine and Animal Productions, University of Naples “Federico II”, 80137 Naples, Italy; sara.damiano@unina.it

**Keywords:** leukemia, marine organisms, marine biotechnology, marine natural products, anticancer

## Abstract

According to the WHO classification of tumors, more than 150 typologies of hematopoietic and lymphoid tumors exist, and most of them remain incurable diseases that require innovative approaches to improve therapeutic outcome and avoid side effects. Marine organisms represent a reservoir of novel bioactive metabolites, but they are still less studied compared to their terrestrial counterparts. This review is focused on marine natural products with anticancer activity against hematological tumors, highlighting recent advances and possible perspectives. Until now, there are five commercially available marine-derived compounds for the treatment of various hematopoietic cancers (e.g., leukemia and lymphoma), two molecules in clinical trials, and series of compounds and/or extracts from marine micro- and macroorganisms which have shown promising properties. In addition, the mechanisms of action of several active compounds and extracts are still unknown and require further study. The continuous upgrading of omics technologies has also allowed identifying enzymes with possible bioactivity (e.g., l-asparaginase is currently used for the treatment of leukemia) or the enzymes involved in the synthesis of bioactive secondary metabolites which can be the target of heterologous expression and genetic engineering.

## 1. Introduction

Cancer is one of the leading causes of death worldwide [1]. It causes the second highest incidence of death after cardiovascular diseases in industrialized countries accounting for nearly 10 million deaths in 2020 (as reported by the World Health Organization (WHO); https://www.who.int/news-room/fact-sheets/detail/cancer; accessed on 22 April 2021). Cancer includes a large group of pathologies related to the unrestrained proliferation of cells in the body and can also spread into other tissues causing metastases that are often lethal [1]. Although a classification system should be flexible and changeable as new information accumulates, WHO, in collaboration with the Society for Hematopathology and the European Association of Hematopathology, published the classification of tumors of the hematopoietic and lymphoid tissues in 2001 [2]. Seven years later, thanks to new scientific and clinical information accumulation, the classification was updated and published as part of the 4th edition of the WHO monograph series [3]. The combination of several data, including clinical, morphologic, immunophenotypic, and genetic features, and the input of 70 internationally recognized clinicians and clinical scientists, over 150 hematopathologists, clinical hematologists, and scientists allowed the final writing of the new WHO “Classification of Tumors of the Hematopoietic and Lymphoid Tissues”. According to this classification, more than 150 typologies of hematopoietic and lymphoid tumors exist [4]. 

Most of the natural products currently on the market derive from terrestrial sources [5,6], including paclitaxel, derived from the bark of the Pacific yew tree (*Taxus brevifolia*) and in use for the treatment of breast, lung, and ovarian cancer (https://dtp.cancer.gov/timeline/flash/success_stories/s2_taxol.htm; Accessed on 6 May 2021); vinblastine and vincristine, isolated from the Madagascar periwinkle *Catharanthus roseus* and used for the treatment of a variety of cancers [7]; and doxorubicin, an anthracycline antibiotic isolated from *Streptomyces peucetius* var. *caesius* generally used in combination with other drugs for the treatment of breast cancer, Hodgkin’s and non-Hodgkin’s lymphoma, leukemia, bronchogenic carcinoma, gastric carcinoma, sarcomas, and thyroid carcinoma [8]. In recent years, several researchers have focused their attention on vaccines, gene therapies, and treatments with monoclonal antibodies for the study of the treatment of hematological cancers disease with promising results. In particular, the success of a molecular targeting therapy requires defining the correct “molecular target”, selecting the right active drug against a specific “target”, and selecting a group of patients who benefit from treatment. Unfortunately, some patients are particularly sensitive to these drugs and may develop severe toxicity [9]. Moreover, immunotherapy has been shown to be an effective therapeutic approach in the treatment of cancers, including hematopoietic malignancies. Clinical trials have shown encouraging results, but future trials may need to incorporate new targets with greater safety and efficacy to ensure long-term benefits [10]. Recently, the marine environment has received increasing attention and has been proposed as a valuable source of new bioactive natural products (marine natural products (MNPs)) [11,12]. Oceans cover nearly 70% of the Earth’s surface, more than 36,000 [13] marine natural products have been isolated thus far, and 100s of new compounds are discovered every year [14]. Since 2008, about 1000 marine-derived compounds are newly discovered every year [15]. The marine environment is characterized by huge biodiversity of species (about 240,000 known species according to The World Register of Marine Species; http://www.marinespecies.org/; Accessed on 6 May 2021) which have adapted to live in multiple extreme environments, e.g., temperatures from −1.5 °C in polar waters to 350 °C in hydrothermal waters, various depths with an increase of pressure of one atmosphere for every 10 m, nutrient concentrations from low (oligotrophic) to high (eutrophic), light intensities from very high at the surface to completely dark in the deepest places [14,16]. Adaptation strategies to environmental parameter variation, as well as predator pressure or stress exposure, have resulted in the evolution of chemical diversity as well. Marine organisms are known to produce series of compounds, derived from their primary and secondary metabolism, which first show bioactivities at sea, such as antipredator, allelopathic, antimicrobial, and infochemical activities, but, thanks to in vitro and in vivo screenings, have also been proven to have possible applications for the treatment and prevention of human pathologies, such as cancer [11,12,17,18,19,20,21,22,23,24,25]. This review reports the compounds of marine origin actually on the market or in clinical trials reported at https://www.midwestern.edu/departments/marinepharmacology/clinical-pipeline.xml (Accessed on 6 May 2021) and the compounds/extracts/fractions reported in the literature found when searching the keywords “hematopoietic cancer”, “marine organisms”, “lymphoma”, and “leukemia” in the PubMed database.

## 2. Compounds on the Market or in Clinical Trials

Currently, there are five compounds derived from marine organisms commercially available for the treatment of hematological tumors, one in Phase III clinical trials, and one in Phase II (as reported at https://www.midwestern.edu/departments/marinepharmacology/clinical-pipeline.xml, Accessed on 6 May 2021; Table 1 and Table 2).

### 2.1. Marine Compounds Already Commercially Available and Currently in Use

Various MNPs derived from both macro- and microorganisms have shown activities for hematopoietic malignancies, passed clinical trials, and are actually in clinical use. In 1969, the first MNP was approved by the Food and Drug Administration (FDA) for leukemia treatment, which also was the first marine-derived anticancer agent to be developed for clinical use, named cytarabine (Ara-C), and commercialized as Cytosar^®^ by the company Pfizer. The active compound is a synthetic analog of a C-nucleoside isolated from the Caribbean sponge *Cryptotethya crypta* and is in use today to treat acute myelocytic leukemia and non-Hodgkin’s lymphoma [12,26]. Regarding its mechanism of action, cytarabine acts by inhibiting the DNA polymerase. 

Several years later, another compound named brentuximab vedotin (SGN-35), inspired from the cyanobacteria compound dolastatin 10 [27], FDA approved in 2011, was commercialized as Adcetris^®^ by Seattle Genetics for anaplastic large T-cell systemic malignant lymphoma and Hodgkin’s disease. Brentuximab vedotin is an antibody–drug conjugate consisting of a chimeric anti-CD30 monoclonal antibody attached via a valine–citrulline linker to the potent microtubule inhibitor monomethyl auristatin E (MMAE). Brentuximab vedotin’s molecular targets are in fact CD30 and microtubules. The monoclonal antibody cAC10 targets the CD30 antigen on systemic anaplastic large-cell lymphoma and Hodgkin’s lymphoma cells [28]. Once brentuximab vedotin is bound to CD30, it is internalized and MMAE is released [29]. 

Plitidepsin, a cyclodepsipeptide originally isolated from the Mediterranean tunicate *Aplidium albicans*, was approved in Australia in December 2018 for the treatment of multiple myeloma, leukemia, and lymphoma [30]. It is commercialized as Aplidin^®^ by Pharmamar and has as molecular target the translation elongation factor 1A2 (eEF1A2). Recently, White et al. [31] explored plitidepsin possible antiviral activity and showed that it also had potent preclinical efficacy against SARS-CoV-2 by targeting the host protein eEF1A.

In the last two years, two other compounds isolated from marine organisms (mollusk/cyanobacterium) found applications for hematological cancers. Polatuzumab vedotin (DCDS-4501A) was FDA approved in 2019 as Polivy^TM^ (Genetech/Roche) for non-Hodgkin’s lymphoma, chronic lymphocytic leukemia, lymphoma, and B-cell lymphoma [32]. It is an antibody–drug conjugate, with an anti-CD79b conjugated to the microtubule inhibitor monomethyl auristatin E (MMAE) [32]. Recently, belantamab mafodotin-blmf (belantamab mafodotin, BLENREP™) was FDA approved in 2020 for the treatment of relapsed or refractory multiple myeloma in adult patients who have received at least four prior therapies by GlaxoSmithKline [33]. Belantamab mafodotin is a monoclonal antibody–drug conjugate (ADC) which comprises an antibody targeting B-cell maturation antigen (BCMA) conjugated to the microtubule inhibitor monomethyl auristatin F (MMAF). 

### 2.2. Marine Compounds in Clinical Trials

In addition to the drugs already in use, two other compounds are currently in clinical trials, marizomib (salinosporamide A; NPI-0052) in Phase III and CX-2029 (ABBV-2029) in Phase II (Table 2). Marizomib was isolated from a marine bacterium of the genus *Salinospora*, the actinobacterium *Salinispora tropica* [34]. It is considered as one of the second-generation proteasome inhibitors (PIs) [35] and, in particular, it has the 20S proteasome as target [36]. It is currently under investigation in relapsed-refractory multiple myeloma, malignant glioma, and other types of solid tumors [37]. Finally, as reported by the company AbbVie Inc., the compound ABBV-2029 is an anti-CD71 probody–drug conjugate being investigated for the treatment of cancer, including diffuse large B-cell lymphoma (https://www.midwestern.edu/departments/marinepharmacology/clinical-pipeline.xml; accessed on 26 April 2021). In particular, it is reported as an ADC targeting CD71 (transferrin receptor) conjugated with the potent microtubule inhibitor monomethyl auristatin E (MMAE) [38]. Trials are still ongoing.

## 3. Microorganisms

Various studies have reported bioactivities against hematopoietic malignancies from marine microorganisms, such as fungi, bacteria, and microalgae (Table 3). Microorganisms are considered a valuable eco-friendly and eco-sustainable source for drug discovery because they can be cultured in small or large volumes in controlled culturing conditions in photobioreactors, allowing the production of a large biomass for further processing [11,21]. In addition, they are characterized by plastic metabolism and can change compound production depending on the culturing conditions (the so-called “OSMAC approach”, one strain multiple compound) [39,40,41,42]. Several studies have shown that different culturing conditions, harvesting times (growth phase), and triggering protocols may influence the bioactivity (e.g., presence of predators) of the extracts [39,40,41,42,43] and, hence, the production of bioactive metabolites. Various studies have also showed the presence of bioactive compounds from microbes associated to other marine organisms. For example, a chromone, named pestalotiopsone F, was isolated from the fungus *Pestalatiopsis* sp. endophytic to the mangrove plant *Rhizophora mucronata.* Pestalotiopsone F showed cytotoxicity against L5178Y lymphoma cells with EC_50_ value of 9 µg/mL [44]. The alkaloid alteramide A produced by *Alteromonas* sp. (bacteria associated with the pacific sponge *Halichondria okadai* strain) showed anticancer activity in vitro against P388 lymphocytic leukemia, L1210 murine lymphoma with the IC_50_ values of 0.1 and 1.7 µg/mL, respectively [45]. 

Another compound isolated from bacteria is the alkaloid altemicidin produced by a marine strain of the bacterium *Streptomyces sioyaensis* SA-1758 with potent antitumor activity in vitro against L1210 murine leukemia with an IC_50_ value of 0.84 µg/mL [46]. Campas et al. [47] showed that prodigiosin (2-methyl-3-pentyl-6-methoxyprodigiosene) obtained from supernatants of the bacterium *Serratia marcescens* induced apoptosis of B-cell chronic lymphocytic leukemia primary cells (*n* = 32 patients) with an IC_50_ value of 116 ± 25 nM. Prodigiosin induced poly-ADP-ribose polymerase (PARP) cleavage and increased caspase 9 in treated cells. Saha et al. (2006) identified a compound from a marine *Actinobacterium* sp. isolated from the Bay of Bengal tolerant to 200 g NaCl/L with molecular weight of 300.2 and predicted molecular formula of C_20_H_28_O_2_. They tested this compound on human leukemia (HL-60) cells and found that it induced the death of 54% of the cells when tested at 0.05 µg/mL. Plinabulin is a synthetic analog of the diketopiperazine phenylahistin (halimide) isolated from marine and terrestrial *Aspergillus* sp. In 2011, Singh et al. [49] showed that the novel vascular disrupting agent (VDA) plinabulin induced cell death in multiple myeloma cells, (MM.1S, MM.1R-dex resistant), RPMI-8226 (human myeloma cells), and INA-6 (IL-6-dependent) without affecting the viability of normal cells (PBMCs). The authors demonstrated by the 3-(4,5-dimethylthiazol-2-yl)-2,5-diphenyltetrazolium bromide (MTT) assay that plinabulin significantly decreased the viability of all MM cell lines in a time dose-dependent manner (the IC_50_ ranged from 8 to 10 nM). Plinabulin was suggested to induce apoptosis, because from the fluorimetric assay a significant increase in the number of annexin V^+^/PI^−^ cells was evident. 

Liu et al. [50] tested aqueous and organic extracts of 40 cyanobacterial isolates from biofilms, gastropods, brackish water, and symbiotic lichen habitats for apoptosis-inducing activity against rat acute myeloid leukemia cells (IPC-81). A total of 28 extracts showed cytotoxicity and 21 of 28 extracts showed selectivity on IPC-81 cells compared to human embryonic kidney (HEK293T) fibroblasts. In particular, organic extracts L26-O (genus *Anabaena*) and L30-O (genus *Nostoc*) were also able to partly overcome the chemotherapy resistance induced by the oncogenic protein Bcl-2, whereas organic extract L1-O (genus *Calothrix*) overcame protection from the deletion of the tumor suppressor protein p53. Cyanobacteria are already known to produce some potentially active compounds, such as the liver toxins microcystins and nodularins, as well as adenosine, able to induce apoptosis in acute myeloid leukemia (AML) cells. To exclude the activity by these known compounds, the authors studied the cell death morphology to identify microcystin-like activity (found for six aqueous extracts) and confirmed their presence by liquid chromatography–mass spectrometry (LC-MS) analysis. To exclude adenosine activity, they used adenosine deaminase to convert adenosine to inosine. Among the active extracts, L26-O had the highest apoptosis-inducing activity, and it also showed activity against the human leukemia patient-derived cell line Molm13. They concluded that different anti-leukemic compounds may be produced by cyanobacteria and further chemical analyses may help to characterize their structures. Oftedal and co-workers [51] also tested the anticancer properties on acute myeloid leukemia HL-60 cells of 41 marine cyanobacterial strains collected from sediment, sand, surface of stones, rocks, water plants, macroalgae, mussels, or mollusks from the Baltic Sea at Porkkala (Gulf of Finland). More than half of the tested species (at a concentration of 4 mg dry weight/biomass/mL) showed apoptotic activity with caspase activation and sensitivity to the recently detected chemotherapy-resistance-associated prosurvival protein LEDGF/p75. 

Mondal et al. [71] reported in a recent review the potential anticancer metabolites derived from cyanobacteria and microalgae, and some of these compounds were reported to have activity against leukemia cells. Coibamide A [52] is a potent depsipeptide isolated from *Leptolyngbya* sp., a Panamanian marine cyanobacterium with a strong cytotoxic activity against HL-60 cells. Medina et al. [52] also showed the decrease of cell cycle at G1 phase with the IC_50_ value of 7.4 nM. Somocystinamide A isolated from the filamentous cyanobacterium *Lyngbya majuscule* is a lipopeptide described in 2008 by Wrasidio W. et al. [53]. It exhibited antiproliferative activity against Jurkat (T cell leukemia), CEM leukemia (T lymphoblast), Molt4T leukemia, and U266 myeloma cells. The authors demonstrated that the compound induced the increase of apoptosis through the activation of caspase 8 with IC_50_ values of 3, 14, 60, and 5.8 µM, respectively. Cryptophycin 1, a macrolide depsipeptide isolated from *Nostoc* sp. (blue green algae/cyanobacterium), showed anticancer activity against L1210 murine leukemia cells as binding with tubulin triggers the disruption of microtubule assembly [54,72]. Lagunamides A–C are other cyclic depsipeptides extracted from the marine cyanobacterium *Lyngbya majuscule.* These compounds exhibited potent cytotoxicity against murine leukemia cell line P388 by causing an increase of cytotoxicity with IC_50_ values of 6.4, 20.5, and 2.1 nM, respectively [55,56]. The lipopeptide microcolin A was also isolated from the *L. majuscula* and induced apoptosis in P388 cells (IC_50_ 380 nM) [57]. Tolytoxin, a microlide described by Patterson [58], was isolated from the lyophilized cells of the cyanobacterium *Seytonema ocellatum*; it exhibited cell growth inhibition of a panel of mammalian cells, including L1210 murine leukemia cells with an IC_50_ value of 3.9 nM. Finally, calothrixins A and B are phenanthridine alkaloids isolated from the marine cyanobacterium *Caothrix* sp. Khan et al. [59] showed that calothrixins A and B were able to inhibit CEM leukemia cell (human T-cell leukemia) proliferation by inducing cell cycle arrest in the G1 and G2/M phases with IC_50_ values of 0.20–5.13 µM. 

Regarding microalgae, Prestegard et al. [60] tested the extracts of ten microalgae isolated by dilution series of samples from microbial biofilms on rocks, sediments, and marine plants in the intertidal zone along fjords on the western part of Norway. Extracts deriving from species belonging to the genera *Melosira*, *Amphora*, *Phaeodactylum,* and *Nitzschia* were found active (more than 30% cell death at 1.5 mg/mL) against IPC-81 rat leukemia cells and were all found to induce leukemia cell death, with either classical apoptotic or autophagic features (seen by morphological analysis by transmission electron microscopy). Successively, MeOH extracts of the dinoflagellates *Amphidinium operculatum* and *Ostreopsis ovata*, collected near Jeju Island (Korea), have been reported to have anticancer activity against HL-60 leukemia cells (at 50 µg/mL) [61]. Another *Amphidinium* species, *A. carterae* (chloroform fraction), as well as the flagellate *Chlorella ovalis* (ethyl acetate fraction) showed anticancer properties against HL-60 cells at both 25 and 50 µg/mL [62]. 

Atasever-Arslan et al. [63] also tested various microalgae (i.e., *Haematococcus pluvialis*, *Scenedesmus obliquus*, *Chlorella vulgaris*, *Spirulina platensis*, *Closterium acerosum*, *Neochloris oleoabundans*, *Microcystis aeruginosa*, *Nannochloropsis oculata*, *Stichococcus bacillaris*, *Klebsormidium flaccidum*, *Botryococcus braunii*, *Ulothrix acuminata, Phaeodactylum tricornutum*, and *Synechococcus elongates*) for possible anti-leukemic activity against HL-60 (human promyelocytic leukemia cell line) and K562 (human chronic myeloid leukemia cell line) cells. *S. bacillaris*, *P. tricornutum*, *M. aeruginosa*, and *N. oculata* extracts were found active against one or both HL-60 and K562 cells (tested concentrations 1–500 µg/mL). *S. bacillaris*, *M. aeruginosa*, and *N. oculata* extracts induced apoptosis in K562 cells, as shown by annexin V, PI, and DNA fragmentation analyses. *P. tricornutum* was more active against HL-60 cells and induced apoptosis, as confirmed by DNA fragmentation, decrease of the phospho-Akt1 protein and cleaved-caspase 3, and increase in cleaved-PARP and Bad proteins. *S. bacillaris* decreased the phospho-Akt1 protein levels and increased phospho-p53 and Bad in HL-60 cells, while *S. bacillaris* increased the phospho-p38 MAPK protein level in K562 cells. In addition, the authors also characterized the composition of oils of these species by gas chromatography–mass spectrometry (GC-MS) and detected 206 different molecules. Of these, only 12 compounds, present in the active extracts were further processed for docking analysis for several key intracellular proteins. The results show that five of these essential oils have interesting possible in silico antileukemic properties (such as similar binding orientation to known inhibitors of key proteins). In particular, the compound 9-Octadecenoic acid, methyl ester, (9Z)- had binding affinity to Akt and Bcl-xL, 2,2,4-Trimethyl-1,3-pentanediyl bis (2-methylpropanoate) to caspase 3, 5,6-Dihydroergosterol to Mdm-2, Tricosane to Mek-1 and PARP-1, and 9-Octadecenamide, (Z)- to p38. 

Chemical analyses showed that the flagellate *Chlorella* contained the compound apigenin [64]. Apigenin, a common dietary flavonoid, was shown to block the proliferation of two types of leukemia, the myeloid and erythroid subtypes, through cell-cycle arrest in the G(2)/M phase for myeloid HL-60 and G(0)/G(1) phase for erythroid TF1 cells. Apigenin inhibited the PI3K/PKB pathway in HL-60 and induced caspase-dependent apoptosis [65]. On the contrary, initiation of autophagy and no apoptosis was observed in TF1 cells. They showed that the common dietary flavonoid apigenin had anticancer activity, but it also may decrease chemotherapy sensitivity, depending on the cell type [65]. Amphidinolide H and carbenolide were both isolated from the dinoflagellate *Amphidinium* sp. GA3P. Amphidinolide H was active against L1210 murine lymphoma cells and also had anti-leukemic activity in vivo in mice implanted with P388 leukemia (in vivo IC_100_ of 0.2 mg/kg) [66]. Carbenolide induced apoptosis in K562 leukemia cells by inhibiting DNA topoisomerase I and II (IC_50_ 30 ng/mL) and increased lifespan in mice implanted with P388 leukemia cells [67].

As described by Samarakoon et al. [68], 8-acetoxy-6-methyloctanoate (NAMO), obtained from the diatom *Phaeodactylum tricornutum*, showed anticancer activity against human promyelocytic leukemia cell line (HL-60), and the highest growth inhibitory activity of about 70% was observed when tested at 50 µg/mL. The authors demonstrated that NAMO induced DNA damage and increased apoptotic body formation with the activation of the pro-apoptotic protein Bax, the suppression of the anti-apoptotic protein Bcl-xL, and an increase of caspase 3 and p53 proteins [68]. The lipid hasla-6(17),9,13,23-tetraene was also isolated from another diatom, named *Haslea ostreria*, and was found to have inhibitory activity on P388 leukemia cells (IC_50_ of 3.3 µg/mL) [69].

Recently, Miceli et al. [70] tested various microalgae, the dinoflagellates *Amphidinium carterae* and *Alexandrium tamarense*, the green alga *Dunaliella salina* and *Tetraselmis suecica*, and the diatoms *Skeletonema japonicum*, *Skeletonema marinoi*, *Chaetoceros affinis*, and *Thalassiosira rotula* against hematological cancer cell line U937 (histiocytic lymphoma). *D. salina*, *T. suecica*, *S. marinoi*, *C. affinis*, and *T. rotula* showed a dose-dependent cytotoxic activity against U937 cells, with *S. marinoi* being the most active species. They showed that monoacylglycerides (MAGs) isolated from the diatom *Skeletonema marinoi* have selective cytotoxic activity against U937 cells compared to normal human mesenchymal progenitor model MePR-2B cell. Chemical analyses (LC-MS) of the raw extract showed that MAGs occurred as 2-monoacyl derivatives and mainly included C16 and C20 analogs. However, these were converted into the corresponding 1-isomers during the purification processes. Pure synthetic 1-monoarachidonoylglycerol was tested on U937 cancer cell line, and the results show that it induced cell death via apoptosis, through caspase 3/7 activation. 

## 4. Macroorganisms

In addition to the active compounds isolated from macroorganisms and currently on the market or clinical trials, other species have shown activities against hematological cancers (Table 4). However, the compounds responsible for the observed activities and/or the mechanisms of action are often still unknown. As reported by Cragg et al. [73], the National Cancer Institute tested over 90,000 extracts of terrestrial and marine plants and invertebrates on its prescreen platform of 60 human cell lines (including K562 and HL-60 leukemia cell lines). Among the marine organism phyla tested, there were Annelida, Bryozoa, Chlorophyta, Chordata, Cnidaria, Cyanophyta, Echinodermata, Mollusca, Phaeophyta, Porifera, Rhodophyta, and Tracheophyta (Mangrove), and 620 of 9945 organisms were found to have anti-leukemia properties (an extract was considered “active” when it showed 50% or greater cytolytic activity against both the K562 and the HL-60 leukemia cells when tested at 100 µg/mL). Even though most of the active extracts did not exhibit selectivity for the leukemia cell lines and showed varying levels of cytotoxicity against other cancer cell lines (e.g., colon, central nervous system, and breast), some Porifera demonstrated selective activity against leukemia cell lines.

Various carotenoids have been shown to have anticancer properties. In particular, fucoxanthin and fucoxanthinol, extracted from brown seaweed *Cladosiphon okamuranus Tokida*, have been shown to decrease cell viability in human primary effusion lymphoma BCBL-1 and TY-1 cells [74]. They arrested cell cycle during the G1 phase and induced caspase-dependent apoptosis, inhibiting anti-apoptotic proteins, nuclear factor-κB (NF-κB), activator protein-1 (AP1), and phosphatidylinositol 3-kinase/Akt pathways. In addition, in vivo studies showed that fucoxanthin reduced the growth of primary effusion lymphoma cells in xenografted mice. Fucoxanthin isolated from the brown alga *Undaria pinnatifida* and *Ishige okamurae* also showed anticancer properties against human leukemic HL-60 cells, with concentrations of 11.3–45.2 and 12.1 μM, respectively [75,76]. Fucoxanthin from *Ishige okamurae* showed increase in reactive oxygen species (ROS), cleavage of caspases 3 and 7 and poly-ADP-ribose polymerase (PARP), and a decrease of Bcl-xL levels in HL-60 cells [76]. Ganesan et al. showed that fucoxanthin, astaxanthin, siphonaxanthin, neoxanthin, and violaxanthin showed anticancer properties against HL-60 cells. The carotenoid siphonaxanthin, isolated from the green macroalga *Codium fragile,* was the most potent carotenoid and showed potent inhibitory effects against human HL-60 cells line (20 µM), by decreasing the anti-apoptotic protein Bcl-2 expression, upregulating the expression of the apoptosis regulators GADD45α (Growth arrest and DNA damage-45 alpha) and DR5 (Death receptor 5), and activating caspase 3 [77]. 

Adult T-cell leukemia (ATL) is a fatal malignancy of T lymphocytes caused by human T-cell leukemia virus type 1 (HTLV-1) infection and is still incurable. Ishikawa et al. [78] tested anticancer properties of fucoxanthin and its metabolite, fucoxanthinol, from the brown algae *Undaria pinnatifida*. Both compounds reduced cell viability of HTLV-1-infected T-cell lines and ATL cells, induced cell cycle arrest during the G(1) phase (by reducing the expression of cyclin D1, cyclin D2, CDK4, and CDK6), induced the expression of the apoptosis regulator protein GADD45α, and reduced the expression of Bcl-2, XIAP, cIAP2, and survivin. In addition, they induced the activation of caspases 3, 8, and 9 and suppressed IkappaB alpha phosphorylation and Jun D expression, resulting in inactivation of nuclear factor-kappaB (NF-κB) and activator protein-1 (AP-1). In vivo experiments, using mice inoculated with HTLV-1-infected T cells, responded to treatment with fucoxanthinol with suppression of tumor growth [78]. Both halocynthiaxanthin and fucoxanthinol, carotenoids isolated from the sea squirt *Halocynthia roretzi*, inhibited the growth of HL-60 cells in a dose- and time-dependent manner. Exposure of HL-60 cells with 12.5 µM of halocynthiaxanthin and fucoxanthinol induced DNA fragmentations and apoptosis (reduction of expression levels of apoptosis-suppressing protein Bcl-2) [79]. Fucoxanthin was also isolated from marine diatoms, such as *Chaetoceros* sp., *Cylindrotheca closterium*, *Odontella aurita*, and *Phaeodactylum tricornutum* [114,115,116].

Brown seaweeds’ (*Laminaria angustata* var. *longissima*, *Laminaria japonica*, *Laminaria japonica* var. *ochotensis*, *Ecklonia cava*, and *Eisenia bicyclis*) antitumor activity was evaluated against L1210 leukemia in mice. Preliminary chemical analyses suggested sulfated polysaccharides as possible responsible of the activity. Hence, Yamamoto et al. [80] prepared crude fucoidan sulfated polysaccharide fractions from *Laminaria angustata* var. *longissima, Laminaria japonica, Laminaria japonica* var. *ochotensis, Ecklonia cava*, and a partially purified fucoidan from *Eisenia bicyclis* and tested their activity (100–200 mg/kg/d for 6 days) in male CDF1 mice inoculated intraperitoneally with a suspension of 10^5^ L1210 cells. Increase in life span of the experimental animals over the controls was observed [80]. All of the fractions, with the exception of the crude fucoidan fraction of *L. japonica*, were active. Finally, the cyclic ether thyrsiferyl 23-acetate was isolated from the red alga *Laurencia obtusa*, while the quinone stypoldione from the alga *Stypopodium zonale* [57,117]. Thyrsiferyl 23-acetate induced apoptosis in P388 and Jurkat leukemia cells by causing DNA fragmentation and chromatin condensation (ED_50_ 0.3 ng/mL), while stypoldione had an effect against P388 cells, both in vitro and in vivo, and inhibited the polymerization of microtubules during initiation of assembly (IC_50_ of 22 µM).

A seashell protein haishengsu (isolated from *Tegillarca granosa* L.) was found to have anticancer properties [82], and Li et al. [81] evaluated whether the addition of haishengsu to the conventional chemotherapies (administered intravenously 2.4 mg in 250 mL normal saline and given daily over 4 h for 28 days) would increase chemosensitivity and improve quality of life in patients with acute leukemia. Patients (248) with acute leukemia were enrolled in a double-blind, placebo-controlled study. In addition to conventional chemotherapy, 142 patients received haishengsu, while 106 received placebo. The complete remission rates in the HSS treatment group were all higher than in the placebo group with non-relapsing leukemia and relapsed leukemia (*p* < 0.05). In vitro studies on a drug-resistant leukemia cell line (K562/ADM cells) showed that the expression of P-gp and sorcin in the haishengsu-treated cells (10–40 µg/mL) were lower than in the control group cells (*p* < 0.01), suggesting this expression inhibition as one of the mechanisms involved in the beneficial effects of haishengsu.

Orostanal, a sterol derivative isolated from the marine sponge *Stelletta hiwasaensis*, and its synthetic analog KPN-2001 induced apoptosis in human acute promyelotic leukemia cell HL-60 at 10 µg/mL (IC_50_ values of 1.7 and 2.2 µM, respectively) [83]. Chen et al. [84] showed that heteronemin, a sesterterpene derivative and the most abundant secondary metabolite in the sponge *Hippospongia* sp., had potent anticancer activity against leukemia cells (EC_50_ values of 0.41 ± 0.08 mg/mL for chronic myelogenous leukemia K562 cells, 0.16 ± 0.05 mg/mL for human promyelocytic leukemia HL-60 cells, and 0.10 ± 0.004 mg/mL for human acute lymphoblastic leukemia Molt4 cells). In particular, they found that heteronemin induced apoptosis in leukemia Molt4 cells by inducing an increase in oxidative stress and mitochondrial dysfunction. In addition, heteronemin upregulated talin and phosphorylated talin expression. Chen and colleagues [84] also evaluated in vivo the effects of heteronemin (0.31 µg/g) on a xenograft nude mice model inoculated with Molt4 cells showing a reduction of tumor volume. Saikia et al. [118] evaluated the chemosensitization of HL-60 cells using heteronemin towards cytarabine chemotherapy. Heteronemin could effectively sensitize HL-60 cells resulting in synergistic toxicity and apoptosis induction. In addition, the authors showed that Heteronemin had farnesyl transferase inhibitory activity and downregulated cytarabine-induced activation of Mitogen-activated protein kinase (MAPK), Activator protein 1 (AP-1), nuclear factor-κB (NF-κB), and c-myc, the down-stream targets of Ras signaling. 

Li et al. isolated from the sponge *Rhabdastrella globostellata* (collected in South China Sea) nine new isomalabaricane-derived compounds, named globostelletins A–I, jaspolides F, rhabdastrellic acid A, (-)-stellettin E, and stellettins C and D. Of these, rhabdastrellic acid A was found to damage the ubiquitin-proteasome pathway (UPP), the central protein degradation system in eukaryotic cells and a potential target for cancer therapy, and induced caspase 3 in HL-60 cells at 5 µM [85]. Smenospongine, a sesquiterpene aminoquinone isolated from the marine sponge *Dactylospongia elegans*, was found to induce erythroid differentiation and G1 phase arrest of K562 chronic myelogenous leukemia cells and apoptosis in HL-60 human acute promyelocytic leukemia cells and U937 human histiocytic lymphoma cells (at 5–15 µM) [86]. In particular, smenospongine induced an increased expression of p21 and inhibited phosphorylation of Rb in K562 cells. Song et al. [90] reported the isolation and structural determination of chujamides A and B, new cyclic cysteine bridged peptides from the sponge *Suberites waedoensis*. These proline-rich dodeca- and undeca-peptides exhibited weak cytotoxicity toward the K562 cell lines with the LC_50_ values of 37 µM for compound A and 55.6 µM for the compound B. Kim et al. [91] collected another sponge species named *Coscinoderma sp.* near Chuuk Island, Micronesia, whose organic extract exhibited moderate cytotoxicity against K562 leukemia cell lines. Especially compounds 1–8 displayed moderate cytotoxicities (LC_50_ of 0.9–5.5 µM). In 2013, Woo et al. [92] isolated bioactive metabolites from sponges in Korean waters, and they reported, for the first time, gombamide A, a cyclic thiohexapeptide from the sponge *Clathria gombawuiensis.* They observed that this highly modified peptide exhibited cytotoxicity against K562 (human leukemia cell lines) with an LC_50_ value of 6.9 µM. 

Halichondrin B, a compound with in vitro and in vivo anticancer activities against murine melanoma and leukemia, was found in various sponges, including *Halichondria okadai*, *Axinella* sp., *Phakellia carteri*, and *Lissondendryx* sp. [119]. Two macrocyclic ketone analogs of halichondrin B, ER-076349 and ER-08652, have also been shown to have antiproliferative activities against various cancer cell lines (Colo 205 and DLD-1 colon cancer, HL-60 promyelocytic leukemia, U937 histiocytic lymphoma, and LNCaP and DU 145 prostate cells) [93]. The mechanism of action of these agents was indistinguishable from the microtubule-destabilizing effects of halichondrin B. Aoki et al. [87,88,89] isolated, from the Indonesian marine sponge *Haliclona* sp., the acetylenic alcohols lembehynes A–C. Dzhemileva et al. [120] tested in vitro the cytotoxic activity of lembehyne B against the immortalized line of human T lymphocyte cells Jurkat and human leukemia cells HL-60 and K562 (IC_50_ values of 2, 2.2, and 3 µM, respectively). Lembehyne B induced apoptosis in all tested cell lines, estimated by detection of phosphatidylserine externalization on the plasmatic membrane after treatment. 

In addition, several sponge-derived peptides have been identified as active against various hematopoietic cancer cell lines [121]. In particular, Phakellistatins 1, 2, 3, and 14 isolated from the sponge *Phakellia* sp. were active against P388 cells with ED_50_ values of 7.5, 0.34, 0.33, and 5 µg/mL, respectively [94,95,96,97]. Criamides A and B from *Cymbastela* sp. and kapakahine B from *Cribrochalina olemda* were also active on P388 cells (ED_50_ of 0.007 and 5 µg/mL, respectively) [98,99]. Theonellamides A–F, isolated from the sponge *Theonella* sp., were active on both P388 and L1210 cell lines with IC_50_ values of 0.9–5 µg/mL [100]. Polytheonamides A–C from *Theonella swinhoei* were active against L1210 cells with EC_50_ value of <4 ng/mL [101], while wainunuamide from *Stylotella aurantium* was active on K562 cells at 18.36 µg/mL [102]. Finally, jaspamide from the sponges *Jaspis* sp. and *Hemiastrella* sp. was active on HL-60 cells at 100 nM, inducing apoptosis and the neutral endopeptidase (NEP)/CD10 expression on the surface of the apoptotic cells [103].

Ascididemin is an alkaloid isolated from the Mediterranean ascidia *Cystodytes dellechiajei* as well as from Okinawan tunicate *Didemnum* species [104]. This compound is known to have anticancer activities [122,123], equally for drug-sensitive and multidrug-resistant leukemia cell lines [122]. Dassonneville et al. [104] showed that that topoisomerases are its main cellular targets. The authors performed relaxation assays using supercoiled DNA and showed that ascididemin stimulated double-stranded cleavage of DNA by topoisomerase II promoting DNA cleavage at sites having a C on the 39 side of the cleaved bond (21 position). Ascididemin strongly induced apoptosis in human HL-60 (EC_50_ value of 0.48 µM) and murine P388 leukemia cells mediated by caspase 3. Activity was evaluated on both P388 and P388CPT5 (sensitive and resistant to camptothecin, respectively) murine leukemia cell lines, and the results show EC_50_ values of 2.4 and 0.05 µM, respectively. In addition, cell cycle analysis showed with that ascididemin treatment there was a loss of cells in the G1 phase accompanied with a large increase in the sub-G1 region. Two other compounds, rossinones A and B, were isolated from an *Aplidium* ascidian species collected from Ross Sea (Antarctica) by dredging at a depth of 200 m. Of these two meroterpenoids, rossinone B exhibited antileukemic activity (against P388 murine leukemia cell-line with an IC_50_ of 0.084 µM), while rossinone A was less active (IC_50_ of 0.39 µM) [105].

As reported by Sperlich et al. [106], the pseudopterosin family, isolated from the soft coral *Antillogorgia elisabethae* (formerly *Pseudopterogorgia elisabethae*), is characterized by secondary metabolites with a variety of biological activities, such as anti-inflammatory, analgesic, wound-healing, and neuromodulatory activities. Pseudopterosins also represent the first commercially licensed marine natural product for use in cosmetic skin care. Sperlich and co-authors [106] showed that pseudopterosins were able to block the inflammatory nuclear factor-κB (NF-κB) signaling pathway by inhibiting the phosphorylation of p65 in leukemia cells, as well as inducing a nuclear translocation of the glucocorticoid receptor and reducing the production of the pro-inflammatory cytokines, such as interleukin-6 (IL-6), tumor necrosis factor alpha (TNF-α), and monocyte chemotactic protein 1 (MCP-1) in THP-1 acute monocytic leukemia cells (IC_50_ value of 24.4 µM).

Bryostatin 1, originally identified in the 1960s, has been evaluated in several Phase I and II clinical trials for the treatment of various cancers (leukemias, ovarian cancers, and prostate cancers) alone or in combination with other drugs (e.g., paclitaxel) [124]. Bryostatin 1 was administrated (by 72 h continuous infusion every 2 weeks at a dose of 120 µg/m^2^ per course) to 25 patients with relapsed, low-grade non-Hodgkin’s lymphoma or chronic lymphocytic leukemia (CLL) and induced the upregulation of the co-expression of CD11c/CD22 on CD20^+^ cells [107]. In addition, bryostatin 1 has also been reported to modulate protein kinase C (PKC) [125]. Discussions are ongoing on whether bryostatin is produced by the bryozoan *Bugula neritina* or the associated bacteria. In fact, an entire putative bryostatin gene cluster from a γ-proteobacterium *Candidatus Endobugulasertula* symbiont of the bryozoan *Bulgua neritina* was discovered via a metagenomic approach [124,126]. 

Triterpene glycosides from sea cucumbers exert a wide spectrum of cytotoxic effects on different cancer cell lines. The triterpene glycoside colochiroside A from *Cercodemas anceps* inhibited the growth of P388 and HL-60 [108] with IC_50_ values of 3.61 ± 0.55 µg/mL; 17-dehydroxyholothurinoside A and griseaside A from *Holoturia grisea* showed cytotoxic activity on human acute lymphoblastic leukemia cell line (Molt-4) with IC_50_ values of 0.34 and 0.521 µM [109]; fuscocinerosides A–C, pervicoside C, and holoturin A from *Holoturia fuscocinerea* showed activity against human leukemia HL-60 (IC_50_ values of 0.88 µg/mL) [109]; philinopsides A and B from *Colochirus quadrangularis* were active on P388 cell line with IC_50_ of 0.60–3.95 µM [110]; pentactasides I–III from *Pentacta quadrangularis* affected several malignant cell lines including P388, with IC_50_ varying from 0.60 to 3.95 µM [111]; and stichoposide C from *Thelenota anax* showed dose-dependent (0.3–1.5 µmol/L) apoptosis induction in human leukemia and colorectal cancer cells following activation of FAS (member of the tumor necrosis factor-receptor [127]) and caspase 8, cleavage of Bid (BH3 interacting-domain death agonist), mitochondrial damage, and caspase 3 activation [112].

Somers et al. [128] fed groups of mice with three different diets for 10 weeks: (1) marine fish (58% homogenized Atlantic smelt and herring); (2) freshwater fish (58% smelt and alewife from the North American Great Lakes); and (3) commercial dry rodent chow. Between 1 and 15 weeks following dietary treatment, 19.4% mice (20/103) unexpectedly developed spontaneous lymphoma. They observed that the disease incidence was mainly when the mice were 7–8 months old and was not equally distributed across treatment groups (the incidence was higher for Groups 2 and 3). Their results suggest that consumption of fat-rich Atlantic smelt and herring protected mice against hematopoietic tumor development [128]. Other in vitro and in vivo (dogs and mice) studies reported the effects of fish oil on hematological neoplasias [129], showing an increase in apoptosis, DNA fragmentation, delay in lymphoma progression, and reduction in metastatic potential. Lipids from fishes have been shown to exert several bioactivities for human health, including brain health [130], neurodegenerative and cardiometabolic diseases [131], and cancer [132]. In addition, it was shown that alkylglycerols (AKG), glycerol ether lipids, isolated from the shark *Centrophorus squamosus*, were able to increase cytosolic calcium rate influx in Jurkat T-cells (T leukemia cells) modulating the permeability of calcium channels [133].

## 5. Multi-Omics Approaches for Drug Discovery 

With the advent of omics technologies, such as genomics and transcriptomics, it is now possible to identify enzymatic pathways which may be responsible for the new active compounds or the active enzymes themselves [134,135,136,137,138,139]. This omics approach is very useful to identify sequence coding enzymes that can be silent in the experimental conditions analyzed but can be activated upon stimulation, such as exposure to stressful conditions. Regarding leukemia treatment, the most studied enzyme is l-asparaginase (E.C. 3.5.1.1), which acts on l-asparagine and produces l-aspartate and ammonia [140]. l-asparaginase is currently used for the treatment of acute lymphoblastic leukemia, acute myeloid leukemia, and non-Hodgkin’s lymphoma [141]. These malignant cells have reduced capacity to produce asparagine synthetase and rely on asparagine supplied directly from the blood. By limiting the supply of asparagine, leukemia cell growth can be inhibited [142,143]. 

l-asparaginases have been found in different microorganisms, including bacteria, fungi, and yeast [144,145]. Screening of marine l-asparginase producing isolates (e.g., for bacteria and fungi) was performed in different studies, e.g., by using the Czapek–Dox or M9 media, containing l-asparagine as the sole nitrogen source, combined with phenol red, which is used as a pH indicator, by measuring the amount of ammonia or aspartic acid released during the reaction or by following the depletion of asparagine, as well as from genome mining and transcriptome analyses. These approaches have allowed the identification of l-asparaginase from different marine microorganisms, such as the bacteria *Bacillus cereus*, *Bacillus pumilus*, *Pseudomonas aeruginosa*, *Enterobacter hormaechei*, *Streptomyces albidoflavus*, *Streptomycetes parvulus*, and *Nocardiopsis alba* and the fungi *Fusarium* sp., *Trichoderma viride*, *Aspergillus* sp., and *Beauveria bassiana* (as reviewed in [140]).

Regarding microalgae, l-asparaginase enzymatic activity was found in the green algae *Chlorella vulgaris* [142], while sequences coding l-asparaginase have been found for the diatoms *Phaeodactylum tricornutum*, *Fragilariopsis cylindrus,* and *Thalassiosira pseudonana*, the flagellate *Nannochloropsis gaditana,* and the dinoflagellate *Amphidinium carterae* [146]. For instance, the transcriptome sequencing of *A. carterae* allowed identifying for the first time in a dinoflagellate a sequence coding l-asparaginase [146]. The in silico identification of the sequences should be combined with heterologous expression and bioactivity screening to confirm the presence of the bioactive enzyme.

In addition to genomic and transcriptomic, proteomic and metabolomics approaches may be useful to identify new peptides and molecules with anticancer activity, and the combination of multiple approaches is helpful in clarifying the compounds, their bioactivities, and the biosynthetic pathways. System biology and synthetic biology will give a great boost to this fast-growing sector, giving the possibility to identify new compounds against hematopoietic malignancies. Recently, Tangerina et al. [147] studied the metabolites produced by the marine *Streptomyces* sp. BRB081 strain cultivated in six different media over different days (by using the one strain many compounds (OSMAC) approach). They used multiple approaches, combining HPLC-MS/MS (high-performance liquid chromatography, coupled to tandem mass spectrometry) data analyzed through GNPS platform (Global Natural Product Social Molecular Networking platform), which contains a database of thousands of reference mass spectrometry spectra of known secondary metabolites, with genome sequencing and mining to search for biosynthetic gene clusters involved in the synthesis of secondary metabolites (by using the AntiSMASH 5.0 software). Analyses allowed identifying desferrioxamines, fatty acid amides, diketopiperazines, xanthurenic acid, the cyclic octapeptides surugamides, and two potentially new surugamide A analogs. In addition, the MTT assay of all crude extracts showed that the bioactivity was correlated to the same extracts for which surugamides production was observed [147]. Their bioactivity screening was performed on colon adenocarcinoma cell line HCT-116, but this approach may be very useful for drug discovery for all types of cancers.

Finally, venomics has also been used to explore the potential applications of venoms [148]. Components isolated from the Jellyfish *Pelagia noctiluca* venom have been found active on human myelogenous leukemia cells (K562) [22,113]. In particular, both the crude venom and the fractions F1–F3 showed a dose-dependent cytotoxicity at concentrations ranging from 5 to 100 µg/mL (Table 4).

## 6. Conclusions

**Figure 1:**
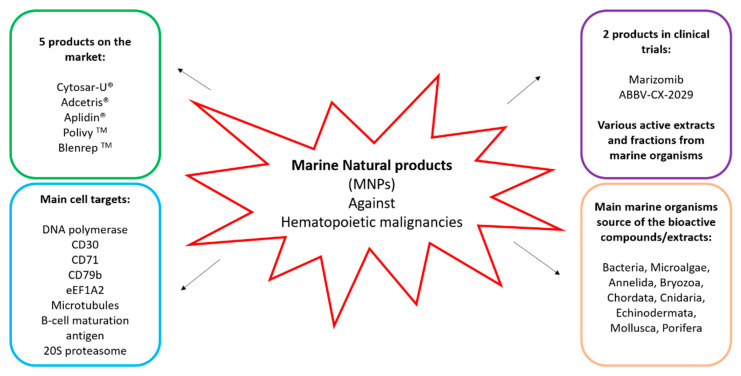
Summary of compounds derived from marine organisms currently on the market or in clinical trials and the known molecular targets.

Availability and easier access to animals and plants on land have favored the search of new natural products from terrestrial organisms in traditional medicine, but there are reports on the use of iodine-rich seaweeds by ancient maritime people, notably the Chinese and Japanese, for their low incidence of goiter [149]. Studies have then shown not only that the marine environment can be the source of bioactive natural products but also that these compounds are in most cases more potent than their terrestrial counterpart, probably because they are diluted in the surrounding water upon release and they need to be potent in order to be effective. Diets rich in marine organisms or their oils have been shown to suppress solid tumor development in humans and rodents, but the possible effects of marine foods on hematopoietic cancers is not yet completely understood [128]. Currently, there are five marine-derived products on the market for the treatment of various hematopoietic cancers (e.g., leukemia and lymphoma), two compounds in clinical trials, and various extracts/fractions which exert interesting bioactivities (Figure 1). This review shows that more than 30 compounds from marine organisms, not in clinical trials, showed in vitro and/or in vivo activities against various hematological cancers representing possible candidates for future drug development. As reported in this review, most of the active compounds against hematopoietic tumors have been isolated from sponges, cyanobacteria, and microalgae. It is difficult to identify the most active compound among them because they have not been tested on the same cell lines or at the same concentrations. Considering the active concentrations found in the literature, among the activities observed at the lowest concentration, there are the *Actynobacterium* sp. (0.05 µg/mL [48]), for which the compound responsible of the activity is still not known, and the compound Ascididemin, isolated from the ascidia *Cystodytes dellechiajei* and the Okinawan tunicate *Didemnum species*, active at 0.48, 2.4, and 0.05 µM against HL-60, P388, and CPT5, respectively [104]. Further studies are necessary to characterize the antitumor activity of the compounds/extracts reported in this review. In fact, in vivo studies, mechanism of action, and identification of the molecular target are missing in several cases. Some of the limitations for this kind of studies are as follows: (1) the supplying of marine organisms, especially uncultivable species; (2) enzymatic pathways for the synthesis of bioactive compounds may be silent in the sampling/culturing conditions, thus genome/transcriptome mining may be useful to identify new molecules by bioinformatics predictions; and (3) there is huge variability among hematopoietic tumor cell lines, hence a compound active for a specific tumor might not be active for others, and vice versa.

Compared to terrestrial organisms, marine environments have been poorly explored. Most studies have focused on marine species collected in temperate and tropical waters, but only 3% of MNPs are derived from polar species [150] and less than 2% from deep-water species [151]. This is due to various reasons, such as sampling difficulties, funding necessity, accessibility, and climate. Even if knowledge related to extreme environments is still limited, recent metagenomics and/or bioactivity screening approaches have demonstrated the biodiversity richness and biotechnological potential of less studied species [16,23,152]. Another recently discussed limitation is the cultivability of some species and reproducibility of the bioactivity. Some species are defined as uncultivable species, but recent efforts have been focused on developing strategies to cultivate the uncultured majority of marine microorganisms, such as the European Union FP7 funded projects BAMMBO (https://cordis.europa.eu/project/id/265896/it; Accessed on 6 May 2021) and MaCuMBA (www.macumbaproject.eu/; Accessed on 6 May 2021), in order to sustainably produce high yields of compounds of interest for the pharmaceutical, cosmetic, and other industrial sectors [153]. Regarding the reproducibility of the bioactivity, laboratory conditions have and are set to mimic the natural environments and/or induce stress by stimulating the production of bioactive compounds, and high-tech fermenters have given great support, especially for microorganisms (e.g., finely regulated tubular, flat plate, twin-layers, inclined tubular, helical, and column photobioreactors) [154]. Considering that the culturing remains not feasible for all organisms, to avoid disruptive collection practices, other approaches are being implemented, such as genomics, transcriptomics, and metabolomics, combined with innovative chemical analyses (including dereplication, structure elucidation, and chemical production [155,156,157]) as well as bioinformatics tools and genetic engineering methodologies. A multidisciplinary approach may represent a successful method to identify and biologically characterize new MNPs for cancer treatment/prevention or as precursors for the generation of new anticancer compounds, possibly being more potent, selective, and with fewer side effects compared to the original molecules. 

## Figures and Tables

**Table 1:** Compounds derived from marine organisms and currently on the market. ADC, antibody–drug conjugate; BCMA, B-cell maturation antigen; eEF1A2, translation elongation factor 1A2; MMAE, microtubule inhibitor monomethyl auristatin E; MMAF, microtubule inhibitor monomethyl auristatin F.

Compound Name	Trademark	Marine Organism	Chemical Class	Molecular Target	Disease Area	Company/Institution
Cytarabine (Ara-C)	Cytosar-U^®^ (1969) *	Sponge	Nucleoside	DNA polymerase	Leukemia	Pfizer
Brentuximab vedotin (SGN-35)	Adcetris^®^ (2011) *	Mollusk/cyanobacterium	ADC (MMAE)	CD30 & microtubules	Anaplastic large T-cell systemic malignant lymphoma, Hodgkin’s disease	Seattle Genetics
Plitidepsin **	Aplidin^®^	Tunicate	Depsipeptide	eEF1A2	Multiple myeloma, leukemia, lymphoma	Pharmamar
Polatuzumab vedotin (DCDS-4501A)	Polivy ^TM^ (2019) *	Mollusk/cyanobacterium	ADC (MMAE)	CD79b & microtubules	Non-Hodgkin’s lymphoma, chronic lymphocytic leukemia, lymphoma, B-Cell lymphoma	Genetech/Roche
Belantamab mafodotin-blmf	Blenrep ^TM^ (2020) *	Mollusk/cyanobacterium	ADC (MMAF)	BCMA	Relapsed/refractory multiple myeloma	GlaxoSmith

* Year Approved by the FDA. ** Approved in Australia in December 2018.

**Table 2:** Compounds derived from marine organisms and currently in clinical trials for the treatment of hematological tumors. ADC, antibody–drug conjugate; MMAE, microtubule inhibitor monomethyl auristatin E.

Compound Name	Marine Organism	Chemical Class	Molecular Target	Disease Area	Company/Institution
**Phase III**					
Marizomib (Salinosporamide A; NPI-0052)	Bacterium	Beta-lactone-gamma lactam	20S proteasome	Cancer: Non-small cell lung cancer, pancreatic cancer, melanoma, lymphoma, multiple myeloma	Triphase
**Phase II**					
ABBV-2029	Mollusk/cyanobacterium	ADC (MMAE)	CD71	Cancer: Solid tumor, head and neck cancer, non-small cell lung cancer, pancreatic cancer, diffuse large B-cell lymphoma	AbbVie & CytomX Therapeutics

**Table 3:** Compounds/extracts from marine microorganisms active for hematopoietic tumors. NA, not available.

Compound	Activity on Cells/Tissues	Organism	Mechanism of Action	Concentration	Reference
Pestalotiopsone F	L5178Y lymphoma cells	Fungus *Pestalatiopsis sp.* endophytic to the mangrove plant *Rhizophora mucronata*	NA	EC_50_ value of 9 µg/mL	[44]
Alteramide	P388 lymphocytic leukemia, L1210 murine lymphoma	*Alteromonas* sp. (bacteria associated with the pacific sponge *Halichondria okadai*)	NA	IC_50_ values of 0.1 and 1.7 µg/mL, respectively	[45]
Altemicidin	L1210 murine leukemia	Bacteria *Streptomyces sioyaensis* SA-1758	NA	IC_50_ value of 0.84 µg/mL	[46]
Prodigiosin (2-methyl-3-pentyl-6-methoxyprodigiosene)	B-cell chronic lymphocytic leukemia primary cells (*n* = 32 patients)	Bacterium *Serratia marcescens*	Apoptosis, induced PARP cleavage, and increased caspase 9	IC_50_ value of 116 ± 25 nM.	[47]
NA	Acute myeloid leukemia HL-60 cells	*Actinobacterium* sp.	NA	0.05 µg/mL	[48]
Plinabulin	Multiple myeloma cells, (MM.1S, MM.1R-dex resistant), RPMI-8226, and INA-6 (IL-6-dependent)	*Aspergillus* sp.	Apoptosis	IC_50_ value of 8–10 nM	[49]
Aqueous and organic extracts	Rat acute myeloid leukemia cells (IPC-81) and human leukemia patient-derived cell line, Molm13	40 cyanobacterial isolates	Apoptosis	NA	[50]
Extracts	Acute myeloid leukemia HL-60 cells	41 marine cyanobacterial strains	Apoptosis	4 mg dry weight/biomass/mL	[51]
Colbamide A	HL-60 cells	Cyanobacterium *Leptolyngbya* sp.	NA	IC_50_ value of 7.4 nM	[52]
Somocystinamide A	CEM (Leukemia) U266 (myeloma)	Cyanobacterium *Lyngbya majuscula*	Apoptosis via caspase 8	IC_50_ values of 14 and 5.8 µM	[53]
Cryptophycim 1	L1210 (murine leukemia cells)	Cyanobacterium *Nostoc* sp.	Disruption of microtubule assembly	NS	[54]
Lagunamidase A,B,C	P388 (murine leukemia cells)	Cyanobacterium *Lyngbya majuscule*	NA	IC_50_ values of 6.4, 20.5 and 2.1 nM	[55,56]
Microcolin A	P388 cells	Cyanobacterium *Lyngbya majuscula*	NA	IC_50_ value of 380 nM	[57]
Tolitoxin	L1210 (murine leukemia cells)	Cyanobacterium *Seytonema ocellatum*	NA	IC_50_ value of 3.9 nM	[58]
Calothrixins A and B	CEM (leukemia)	Cyanobacterium *Colothrix* sp.	G_1_ arrest and cell accumulation in S and G_2/_M phases	IC_50_ values of 0.20–5.13 µM	[59]
NA	IPC-81 rat leukemia cells	Ten microalgae	Classical apoptotic or autophagic features	1.5 mg/mL	[60]
NA	human promyelocytic leukemia cell line (HL-60)	Microalgae *Amphidinium operculatum* and *Ostreopsis ovata*	NA	50 µg/mL	[61]
NA	human promyelocytic leukemia cell line (HL-60)	Microalgae *Amphidinium carterae* and *Chlorella ovalis*	NA	25 and 50 µg/mL	[62]
NA	human promyelocytic leukemia cell line (HL-60) and human chronic myeloid leukemia cell line (K562)	Various microalgae	Apoptosis (see text for details)	Tested concentrations of 1–500 µg/mL	[63]
Apigenin	Human myeloid HL-60 and erythroid TF1 cancer cells.	Microalga *Chlorella* sp., etc.	Inhibited PI3K/PKB pathway/induced apoptosis in HL-60 cells; Autophagy in TF1 cells	30–100 µM	[64,65]
Amphidinolide H	L1210 murine lymphoma cells and in mice implanted with P388 leukemia	*Amphidinium* sp. GA3P.	NA	In vitro: IC_50_ value of 0.5 ng/mL In vivo: IC_100_ value of 0.2 mg/kg	[66]
Carbenolide	K562 leukemia cells	*Amphidinium* sp. GA3P.	Inhibited DNA topoisomerase I and II; increased lifespan in mice implanted with P388 leukemia cells	IC_50_ 30 ng/mL	[67]
8-acetoxy-6-methyloctanoate (NAMO)	human promyelocytic leukemia cell line (HL-60)	Microalga *Phaeodactylum tricornutum*	DNA damage, increased apoptotic body formation with the activation of the pro-apoptotic protein Bax, the suppression of the anti-apoptotic protein Bcl-xL, and an increase of caspase 3 and p53 proteins	50 µg/mL	[68]
Hasla-6(17),9,13,23- tetraene	P388 leukemia cells	Microalga *Haslea ostreria*	NA	IC_50_ value of 3.3 µg/mL	[69]
Monoacylglycerides (MAGs)	U937 (human histiocytic lymphoma)	Microalga *S. marinoi*	Apoptosis; activation caspase 3/7	5–50 µg/mL	[70]
Extracts	U937 (human histiocytic lymphoma)	Microalgae *D. salina*, *T. suecica*, *S. marinoi*, *C. affinis* and *T. rotula*	NA	100–500 µg/mL	[70]

**Table 4:** Compounds/extracts from marine macroorganisms active for hematopoietic tumors. NA, not available.

Compound	Activity on Cells/Tissues	Organism	Mechanism of Action	Concentration	Reference
NA	K562 and HL-60 leukemia cell lines	Annelida, Bryozoa, Chlorophyta, Chordata, Cnidaria, Cyanophyta, Echinodermata, Mollusca, Phaeophyta, Porifera, Rhodophyta and Tracheophyta	NA	100 µg/mL	[73]
Fucoxanthin and fucoxanthinol	Human primary effusion lymphoma BCBL-1 and TY-1 cells	Brown seaweed *Cladosiphon okamuranus Tokida*	Cell cycle arrest during the G1 phase and caspase-dependent apoptosis, inhibited the activation of nuclear factor-κB, activator protein-1, and phosphatidylinositol 3-kinase/Akt pathways, and downregulated anti-apoptotic proteins and cell cycle	IC_50_ values of 2.4–3.3 and 1.1 µM, respectively	[74]
Fucoxanthin	Human leukemic HL-60 cells	Brown alga *Undaria pinnatifida*	NA	11.3–45.2 μM	[75]
Fucoxanthin	Human leukemic HL-60 cells	Brown alga Ishige okamurae	Increase in reactive oxygen species (ROS), cleavage of caspases 3 and 7, and poly-ADP-ribose polymerase (PARP), and decrease in Bcl-xL levels	12.1 μM	[76]
Siphonaxanthin	Human leukemic HL-60 cells	Green macroalga *Codium fragile*	Decreased Bcl-2 expression, upregulated the expression of GADD45α and DR5, and activated caspase 3	20 µM	[77]
Fucoxanthin and fucoxanthinol	Adult T-cell leukemia (ATL), human T-cell leukemia virus type 1 (HTLV-1)	Brown algae *Undaria pinnatifida*	In vitro: Cell cycle arrest during G(1) phase (by reducing the expression of cyclin D1, cyclin D2, CDK4, and CDK6), induced the expression of the apoptosis regulator protein GADD45α, and reduced the expression of Bcl-2, XIAP, cIAP2, and survivin. In addition, they induced the activation of caspases 3, 8, and 9, suppressed IkappaBalpha phosphoryla-tion and JunD expression; In vivo: Suppression of tumor growth	IC_50_ values of 1.20–4.46 μM and 0.86–1.83 μM, respectively.	[78]
Halocynthiaxanthin and fucoxanthinol	Human leukemic HL-60 cells	Sea squirt *Halocynthia roretzi*	DNA fragmentations and apoptosis (reduction of expression levels of apoptosis-suppressing protein Bcl-2)	12.5 µM	[79]
Fucoidan	Male CDF1 mice inoculated intraperitoneally with a suspension of 10^5^ leukemia L1210 cells	Seaweeds *Laminaria angustata* var. *longissima*, *Laminaria japonica*, *Laminaria japonica* var. *ochotensis*, *Ecklonia cava* and *Eisenia bicyclis*	NA	100–200 mg/kg/d for 6 days	[80]
Haishengsu	In patients and drug-resistant leukemia cell line (K562/ADM cells)	Seashell *Tegillarca granosa* L.	Reduction in the expression of P-gp and sorcin	In vivo: 2.4 mg in 250 mL normal saline and given daily over 4 h for 28 days; In vitro: 10–40 µg/mL	[81,82]
Orostal and KPN-2001	Human leukemic HL-60 cells	Sponge *Stelletta hiwasaensis*	Apoptosis	IC_50_ value of 1.7 and 2.2 µM	[83]
Heteronemin	Chronic myelogenous leukemia K562 cells, human promyelocytic leukemia HL-60 cells, human acute lymphoblastic leukemia Molt4 cells; in vivo mice model inoculated with Molt4 cells	Sponge *Hippospongia* sp	Apoptosis in leukemia Molt4 cells by inducing an increase in oxidative stress (increase in reactive oxygen species production), mitochondrial dysfunction, upregulation of talin and phosphorylated talin expression	In vitro: EC_50_ 0.41 ± 0.08 mg/mL for K562 cells, 0.16 ± 0.05 mg/mL for HL-60 cells and 0.10 ± 0.004 mg/mL for Molt4 cells; In vivo: 0.31 µg/g	[84]
Rhabdastrellic acid A	Human leukemic HL-60 cells	Sponge *Rhabdastrella globostellata*	Damages the ubiquitin-proteasome pathway (UPP), induced caspase 3	5 µM	[85]
Smenospongine	Chronic myelogenous leukemia cells K562, HL-60 and human histiocytic lymphoma cells U937	Sponge Dactylospongia elegans	Induced G1 phase arrest and increased expression of p21 and inhibited phosphorylation of Rb in K562 cells	5–15 µM	[86]
Lembehynes A, B and C	Immortalized line of human T lymphocyte cells Jurkat, and human leukemia cells HL-60 and K562	Indonesian marine sponge *Haliclona* sp.	Apoptosis	IC_50_ values of 2, 2.2, and 3 µM, respectively	[87,88,89]
Chujamides A and B	Chronic myelogenous leukemia cells K562	Sponge *Suberites waedoensis*	NA	LC_50_ values of 37 and 55.6 µM, respectively	[90]
Compounds 1–8	Chronic myelogenous leukemia cells K562	Sponge *Coscinoderma sp.*	NA	LC_50_ values of 0.9–5.5 µM	[91]
Gombamide A	Chronic myelogenous leukemia cells K562	Sponge *Clathria gombawuiensis*	NA	LC_50_ value of 6.9 µM	[92]
ER-076349 and ER08652	Human leukemic HL-60 cells and U937 histiocytic lymphoma	Analogs of Halichondrin B originally found in a variety of marine sponges	Microtubule destabilized effects	IC_50_ values of 0.15 and 0.07 nM	[93]
Phakellistatins 1, 2 and 3	P388 murine leukemia cell-line	Sponge *Phakellia costata*	NA	ED_50_ values of 7.5, 0.34, and 0.33 µg/mL, respectively	[94,95,96]
Phakellistatin 14	P388 murine leukemia cell line	Sponge *Phakellia* sp.	NA	ED_50_ value of 5 µg/mL	[97]
Criamides A and B	P388 murine leukemia cell line	Sponge *Cymbastela* sp.	NA	ED_50_ value of 0.007 µg/mL	[98]
Kapakahine B	P388 murine leukemia cell line	Sponge *Cribrochalina olemda*	NA	IC_50_ value of 5 µg/mL	[99]
Theonellamides A– F	P388 and L1210 murine leukemia cells	Sponge *Theonella* sp.	NA	IC_50_ values of 0.9–5 µg/mL	[100]
Polytheonamides A-C	Mouse lymphocytic leukemia L1210 cells	Sponge *Theonella swinhoei*	NA	EC_50_ value of <4 ng/mL	[101]
Wainunuamide	K562 leukemia cancer cells	Sponge *Stylotella aurantium*	NA	18.36 µg/mL	[102]
Jaspamide	Human promyelocytic leukemia HL-60	Sponge *Jaspis* sp. and *Hemiastrella* sp.	Apoptosis, neutral endopeptidase (NEP)/CD10 expression on the surface of the apoptotic cells	100 nM	[103]
Ascididemin	HL-60 and the murine P388 and P388CPT5 (sensitive and resistant to camptothecin, respectively) leukemia cell lines	Ascidia *Cystodytes dellechiajei*/Okinawan tunicate *Didemnum* species	Double-stranded cleavage of DNA by topoisomerase II, apoptosis mediated by caspase 3	In vitro: EC_50_ values of 0.48, 2.4, and 0.05 µM, respectively	[104]
Rossinones A and B	P388 murine leukemia cell-line	*Aplidium* ascidian species	NA	IC_50_ values of 0.39 and 0.084 µM, respectively	[105]
Pseudopterosins,	THP-1 acute monocytic leukemia cells	Soft coral *Antillogorgia elisabethae* (formerly *Pseudopterogorgia elisabethae*)	Block of nuclear factor-κB (NF- κB), inhibiting the phosphorylation of p65, inducing a nuclear translocation of the glucocorticoid receptor and reducing the production of the pro-inflammatory cytokines, such as interleukin-6 (IL-6), tumor necrosis factor alpha (TNF-α), and monocyte chemotactic protein 1 (MCP-1)	IC_50_ value of 24.4 µM	[106]
Bryostatin 1	Patients with low grade non-Hodgkin’s lymphoma or chronic lymphocytic leukemia	Bryozoan *Bugula neritina*	Upregulation in the co-expression of CD11c/CD22 on CD20^+^ B cells.	120 µg/m^2^	[107]
Colochiroside A	P388 and HL-60 leukemia cell-line	Sea cucumber *Cercodemas anceps*	Inhibited cell growth	3.61 ± 0.55 µg/mL	[108]
17-dehydroxyholothurinoside A and griseaside A	Human acute lymphoblastic leukemia cell line Molt-4	Sea cucumber *Holoturia grisea*	Cytotoxicity	0.34 and 0.521 µM	[109]
Fuscocinerosides A–C, Pervicoside C, and holoturin A	Human leukemia HL-60	Sea cucumber *Holoturia fuscocinerea*	Cytotoxicity	IC_50_ value of 0.88 µg/mL	[109]
Philinopsides A and B	P388 murine leukemia cell-line	Sea cucumber *Colochirus quadrangularis*	Cytotoxicity	IC_50_ values of 0.60–3.95 µM	[110]
Pentactasides I–III	P388 murine leukemia cell-line	Sea cucumber *Colochirus quadrangularis*	Cytotoxicity	IC_50_ 0.60 to 3.65 µM	[111]
Stichoposide C	Human leukemia HL-60	Sea cucumber *Thelenota anax*	Activation of FAS and caspase 8, cleavage of Bid, mitochondrial damage, and caspase 3 activation	Dose-dependent, 0.3–1.5 µmol/L	[112]
Raw venom, fractions F1–F3	Human myelogenous leukemia cells (K562)	Jellyfish *Pelagia noctiluca*	Cytotoxicity	5–100 µg/mL	[113]

## Data Availability

Not applicable.
